# Dosimetric and clinical analysis of pseudo-progression versus recurrence after hypo-fractionated radiotherapy for brain metastases

**DOI:** 10.1186/s13014-023-02214-7

**Published:** 2023-02-14

**Authors:** Siran Yang, Yuchao Ma, Yingjie Xu, Qingfeng Liu, Ye Zhang, Xiaodong Huang, Xuesong Chen, Kai Wang, Ruizhi Zhao, Jianping Xiao, Hongmei Zhang

**Affiliations:** 1 Departments of Radiation Oncology, National Cancer Center/National Clinical Research Center for Cancer/Cancer Hospital, Chinese Academy of Medical Sciences and Peking Union Medical College, Beijing, People’s Republic of China; 2 Departments of Diagnostic Radiology, National Cancer Center/National Clinical Research Center for Cancer/Cancer Hospital, Chinese Academy of Medical Sciences and Peking Union Medical College, Beijing, People’s Republic of China; 3 Department of Radiation Oncology, Peking University Shenzhen Hospital, Shenzhen, People’s Republic of China

**Keywords:** Brain metastases, Hypo-fractionated radiation therapy, Pseudo-progression, Tumor recurrence, Dosimetry, Magnetic resonance imaging

## Abstract

**Background:**

The main challenge in follow-up duration of patients with brain metastases after stereotactic radiotherapy is to distinguish between pseudo-progression and tumor recurrence. The objective of this study is to retrospectively analyze the predictive factors.

**Methods:**

The study included 123 patients with enlarged brain metastases after hypo-fractionated radiotherapy in our center from March 2009 to October 2019, and the baseline clinical features, radiotherapy planning parameters, and enhanced magnetic resonance imaging before and after radiation therapy were analyzed. Logistic regression was performed to compare the differences between groups. Independent risk factors with *P* < 0.05 and associated with recurrence were used to establish a nomogram prediction model and validated by Bootstrap repeated sampling, ﻿which was validated in an internal cohort (n = 23) from October 2019 to December 2021.

**Results:**

The median follow-up time was 68.4 months (range, 8.9–146.2 months). A total of 76 (61.8%) patients were evaluated as pseudo-progression, 47 patients (38.2%) were evaluated as tumor recurrence. The median time to pseudo-progression and tumor recurrence were 18.3 months (quartile range, 9.4–27.8 months) and 12.9 months (quartile range, 8.7–19.6 months) respectively. Variables associated with tumor recurrence included: gross tumor volume ≥ 6 cc, biological effective dose < 60 Gy, target coverage < 96% and no targeted therapy. The area under curve values were 0.730 and 0.967 in the training and validation cohorts, respectively. Thirty-one patients received salvage therapy in the tumor recurrence group. The survival time in pseudo-progression and tumor recurrence groups were 66.3 months (95% CI 56.8–75.9 months) and 39.6 months (95% CI 29.2–50.0 months, respectively; *P* = 0.001).

**Conclusions:**

Clinical and dosimetry features of hypo-fractionated radiation therapy based on enhanced brain magnetic resonance can help distinguish pseudo-progression from tumor recurrence after hypo-fractionated radiotherapy for brain metastases. Gross tumor volume, biological effective dose, target coverage, and having received targeted therapy or not were factors associated with the occurrence of tumor recurrence, and the individual risk could be estimated by the nomogram effectively.

**Supplementary Information:**

The online version contains supplementary material available at 10.1186/s13014-023-02214-7.

## Background

With the survival time of patients with advanced-staged malignancies continues to be prolonged, an increasing number of them develop brain metastases (BM) during the disease progression. Based on the development of radiotherapy (RT) techniques and the protection of neurological function, local hypo-fractionated radiotherapy (HFRT) has become the preferred non-surgical treatment option for patients with limited number of BM. HFRT, including stereotactic radiation surgery (SRS) or hypo-fractionated stereotactic radiation therapy (HFSRT), has been applied as an emerging radiotherapy technique for clinical BM treatment in recent years [1–3]. However, post-radiation changes such as the enlargement of the treated lesions can be observed in a significant proportion of the patients after HFRT, but no signs of tumor recurrence (TR) are found after long-term follow-up or surgical resection, which is actually a pseudo-progression (PP) [4, 5].

PP is usually characterized by an increase in the size of BM after radiotherapy, accompanied by enhancement and enlargement of the peritumoral edema area; clinically, it is characterized by deterioration in neurological symptoms, often manifested as radiation necrosis (RN). These features are often difficult to distinguish from TR. However, from another perspective, RN marks the successful disruption of the blood–brain barrier and stimulation of inflammatory cell aggregation by HFRT [6, 7]. Patients with mild PP can be temporarily relieved with dehydration or anti-angiogenic agents [8], but a small proportion of patients who develop severe symptoms rely on invasive treatments such as surgical resection for symptomatic management. However, if the PP areas are re-irradiated, the extent of necrosis and edema may aggravate progressively, with worse neurological symptoms or even tumor rupture or brain herniation, endangering patients' lives. Because of the different therapeutic measures and clinical outcome, it’s turning out to be increasingly imperative to distinguish TR from PP. Imaging physicians need to use patients’ imaging features, clinical information and treatment modalities for diagnosis, and radiation oncologists need to balance the possibility of tumor control and RN during initial radiation therapy planning to avoid under-dosing or over-segmentation of HFRT.

Previous studies focused on functional magnetic resonance imaging (MRI), positron emission tomography (PET), or were limited to the analysis of clinical features or dose parameters of a single fractionated SRS. The aim of this study was to retrospectively investigate patients with BM who underwent HFRT and had long-term enhanced brain MRI imaging follow-up data in our institution, and to explore the possible dosimetric and imaging factors associated with PP and TR, providing data for subsequent clinical practice and prognostic judgment.

## Methods

### Eligibility criteria

The eligibility criteria were as follows: (1) age ≥ 18 years, (2) presence of imaging-measurable BM diagnosed by RANO criteria [9], (3) receiving 2-mm thin-section brain/spinal cord contrast-enhanced MRI scans pre-/during /post-RT, (4) BM treated with HFRT (≥ 2.5 Gy per fraction), (5) follow-up time ≥ 6 months, and (6) at least 3 times post-treatment brain-enhanced MRI imaging data at our institution and showed enlargement of target lesion during the follow-up period. Exclusion criteria: (1) local treatment (surgery, radiotherapy) of the target lesion prior to HFRT, (2) other serious CNS diseases such as cerebral infarction or hemorrhage within 6 months. In addition, due to the long interval between the previous RT and irradiation, which might have little impact on the cumulative dose, the previous WBRT dose with an interval of more than 1 year was not included in the BED calculation in this study.

The protocol of this trial was approved by our institutional ethics review board (approval number: 2021010711263002); given its retrospective nature, the review board agreed to waive the need for informed consent.

### Radiation regimens

RT was delivered by linear accelerator using multiple techniques, including stereotactic radiotherapy (SRT), normal intensity-modulated radiotherapy (IMRT), volumetric arc rotational intensity-modulated radiotherapy (VMAT), and helical tomotherapy (TOMO). Prior to radiotherapy, a large-aperture computed tomography (CT) scan was performed to simulate localization, and a 3 T thin-section brain-enhanced diagnostic MRI or 1.5 T thin-section brain localization MRI was performed with the patient in a supine position, with hands on either side of the body and a fixed frame or head, neck and shoulder thermoplastic film fixation, covering the cranial vault to the mastoid bone level, with a layer thickness of 2 mm. The images were then uploaded to Brian-lab or Pinnacle system for fusion of localized images to guide target area contouring. The target volumes were defined and contoured as follows: the gross target volume (GTV) was defined as the area of equal or high signal enhancement compared to normal brain of BM visible on the T1-weighted- contrasted MRI; the planned target volume (PTV) was defined as in front, back, left and right outgrowth of GTV by 2 mm, and formed by upper and lower copies; CTV-brain was defined as the whole brain, outgrown by 5–8 mm to form PTV-brain; the normal organs at risk (OAR) included brainstem, spinal cord, optical nerves, optical chiasm, lens, pituitary, hippocampi, and the volume of the whole brain except for the target area was generated as the volume of normal brain tissue. Brainstem-planning OAR volume (PRV) was defined as 2-dimensional outgrowth of brainstem by 3 mm. When the lesions were in the proper distance from hippocampus, we would expand it in two-dimensional directions and copied upper & lower layer. But for patients received WBRT concurrent with HFRT or whose lesions closed to hippocampus, we only had naked hippocampus doses constraint. The margins used for GTV (2 mm) and to the whole brain (5-8 mm) were the same for patients received precise HFRT in our center during the decade. Image guidance was performed using megavoltage cone-beam CT (CBCT) before each RT treatment. According to the historical research data of our center, basically, the average number of displacement changes didn’t exceed 2 mm, so all of the displacements were included in the expansion scope of PTV-brain.

When metastatic lesions were located near vital structures (e.g., brainstem) or re-irradiation areas, the total dose or single fractionated dose was decreased, as appropriate, to reduce the risk of serious toxic effects. The median fraction schedule was determined as follows: 50–52.5 Gy/10–15 fractions (f) for large lesions (≥ 5 cc), 32–42 Gy/4–7 f for lesions close to the functional area, 40–45 Gy/10–15 f for lesions within the brainstem, and 10–15 f for oligo-BM lesions (with less than five brain metastases received local HFRT alone without WBRT). 20–36 Gy/1–3 f for single small focus (with diameter of less than 1 cm and not located in vital structures), 40 Gy/20 f for whole brain radiotherapy (WBRT) concurrent with 60 Gy/15–20 f for simultaneous integrated boost (SIB), 30 Gy/10 f for WBRT followed by HFRT. According to ICRU Report No. 83, 95% of the target volume receiving at least 90% of the prescribed dose. Normal tissue dose limits included as follows: brainstem maximum dose (Dmax) ≤ 54 Gy, spinal cord Dmax ≤ 45 Gy, bilateral optic nerve Dmax ≤ 54 Gy, optic chiasm Dmax ≤ 54 Gy, bilateral len Dmax < 9 Gy, and pituitary was evaluated. Some patients had strict limits for brainstem and hippocampi: brainstem mean dose (Dmean) ≤ 35 Gy, bilateral hippocampus Dmax < 16 Gy.

Patients who had intracranial edema would routinely receive steroids in short term at first. Anti-angiogenic drug was applied when symptoms did not resolve significantly.

### Radiation parameters acquisition

According to ICRU reports No. 83 [10] and No. 91 [11], common target area evaluation parameters, target coverage (TC), prescription isodose/target volume ratio (PITV), target area conformation index (CI), homogeneity index (HI), gradient indexes (GI) and dose of OAR were selected. TC is defined as the ratio of target volume (TV) at the prescribed dose (TV_PV_) to TV, based on the formula: *TC* = *TV*_*PV*_*/TV* × *100%*. TC = 1 indicates that TV is totally covered. PITV is the ratio of the volume covered by the prescribed dose line (PV) to the volume of the target area, PITV < 1 indicates that the target volume is not completely covered by the dose, PITV > 1 indicates that the tissue around the target volume is over-covered, PITV close to 1 indicates that the target volume is highly overlapped, using the formula: *PITV* = *PV*/*TV.* PITV is a calculation for the target lesions, not the whole brain; In addition, the prescribed dose of the BM lesion is usually higher than the whole brain dose, so it is calculated according to the formula. CI is based on Paddick's formula: *CI* = *TV*^*2*^_*PV*_/*(TV* × *PV)*. HI is based on the formula: *HI* = *(D*_*2*_*—D*_*98*_*)*/*D*_*50*_ × *100%*, where D2 is the dose received by 2% of the target volume; D98 is the dose received by 98% of the target volume; D_50_ is the dose received by 50% of the target volume. GI is defined as the ratio of the volume enclosed by 50% of the prescribed dose line to the volume enclosed by the prescribed dose line, and the smaller the value, the faster the dose drop and the larger the dose gradient. The dose dropping zone around the BM is defined as the volume of PTV minus GTV, and its mean dose and the volume that received 95% of the prescribed dose (V95) were assessed. Normal brain tissue was analyzed using Dmean and the volume receiving 30 Gy irradiation (V30).

### Imaging data acquisition

Enhanced brain MRI was first conducted 2 months after RT to evaluate the recent efficacy and then regularly performed every 3 months by 3.0 T MRI (GE Medical Systems, Boston, USA). The following sequences and parameters were collected to obtain axial brain imaging information: (1) T1-weighted-MRI (T1WI): TR/TE = 500/20 ms (ms), field of view: 220 × 220, slice thickness: 5 mm (mm), slices: 50, averages: 1, scan time: 1 min (min) 30 s (s). (2) T13D FDPGR-enhanced image (T1-weighted-contrasted MRI, T1WI + C): TR/TE = 2000/2.3 ms, field of view:: 250 × 250, slice thickness: 2 mm, slices: 192, averages: 1, scan time: 4 min 38 s; (3) T2 Flair PROP-weighted image (T2WI Flair): TR/TE = 8000/81 ms, field of view: 220 × 220, slice thickness: 5 mm, slices: 50, averages: 1, scan time: 1 min 36 s; (4) Diffusion-weighted-Imaging (DWI): scan sequence as T2star_FID_EPI, b = 0, 1000. TR/TE = 2910/56 ms, field of view: 220 × 220, slice thickness: 5 mm, slices: 50, scan time: 47 s.

In addition, the patient's symptoms and signs, KPS score, imaging of the primary tumor and follow-up results were also recorded.

### End points and statistical analysis

The study endpoint was TR. The efficacy was evaluated based on the Response Assessment in Neuro-Oncology of Brain Metastases (RANO-BM) criteria: (1) Effective: improvement in neurological examination, negative cerebrospinal fluid (CSF) exfoliative cytology, improvement in central nervous system (CNS) imaging, improvement in symptom evaluation, no or reduced steroid hormone; (2) Stable: stable neurological examination, negative or positive CSF exfoliative cytology, non-progressive CNS imaging, stable symptom evaluation, stable or reduced steroid hormone dose; (3) Progressive: progressive neurological examination, positive CSF exfoliative cytology, significantly progressive CNS imaging, progressive symptom evaluation, stable or increased steroid hormone dose. Disease control rate (DCR) was defined as the probability of not reaching progression status of RANO-BM. Neurologic treatment-emergent adverse event (AEs) were recorded according to the NRG-RTOG Acute and Late CNS Toxicity Criteria.

The gold standard for determining TR or PP is surgical pathology when clinical outcomes cannot be clinically distinguished. TR is manifested by metastatic tumor cells found in the pathologic tissue, and PP is manifested by demyelination, fibrinoid necrosis, and coagulating necrosis of the cerebral white matter, thickening of the vessel wall, vitreous changes, and narrowing and occlusion of the lumen. However, craniotomy is invasive and usually only applicable to the lesions in surface area that easily reached. If craniotomy is not available to obtain pathological results, functional brain MRI or PET-CT can be used, or more than two qualified and experienced diagnostic imaging physicians would be consulted to increase diagnostic reliability of brain MRI. The clinical diagnosis of PP was made if the edema aggravated and CNS symptoms worsen after HFRT but improved with steroids or anti-angiogenic drugs, and the patient was followed up to obtain long-term survival without observing obvious signs of recurrence.

Continuous variable data were expressed as median or mean ± standard deviation, and the restricted cubic spline function was applied to find the best cut-off value. Comparisons between normally distributed data were made with paired t-tests, and non-normally distributed data with Wilcoxon signed-rank tests; different groups were compared using Mann–Whitney U test for continuous variables and the χ2 or Fisher exact method for categorical variables. Survival was calculated using Kaplan–Meier plots, and log-rank test was used to assess for differences. Logistic regression was used to screen for variables to be included in the analysis, with *P* < 0.100 (two-sided) was considered as statistically significant different. Bootstrap repeated sampling was used to internally validate the model, and those predictors with *P* < 0.050 were included in the subsequent Nomogram prediction modeling. And then the internal validation was performed. Statistical analyses were performed using SPSS (version 26.0, IBM Corporation, Armonk, NY) and R software (version 4.0.3, http://www.r-project.org/).

## Results

### Patients characteristics

**Fig. 1:**
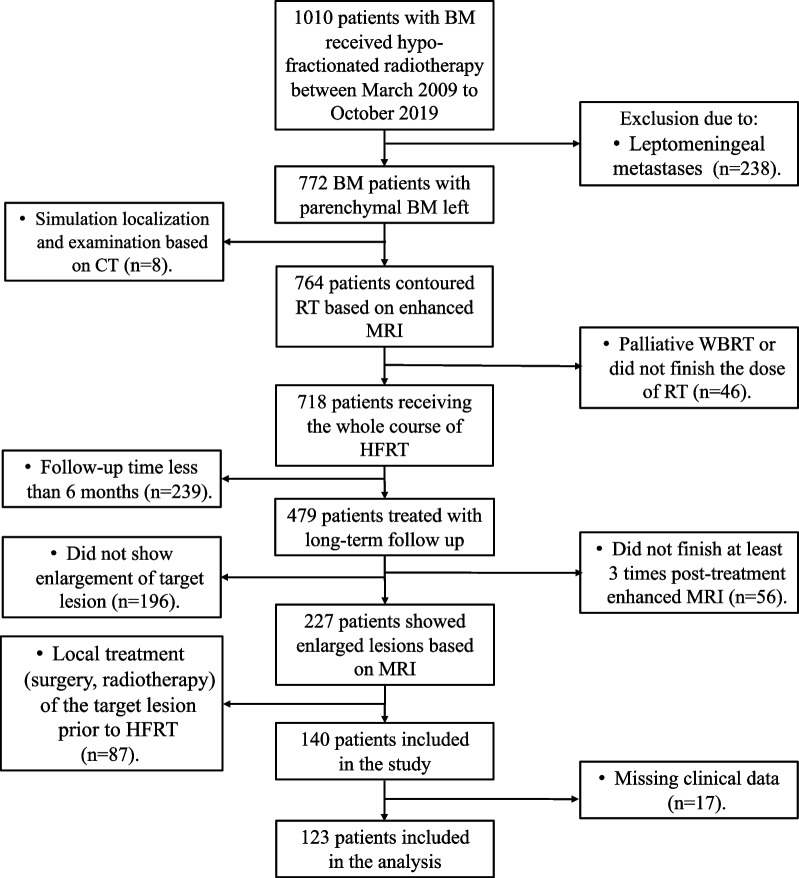
The flow chart of the study

This study included consecutive 138 patients from March 2009 to October 2019 at our institution, with 15 cases excluded due to incomplete data, finally a total of 123 patients were included in the analysis (Fig. 1). Patient baseline characteristics are listed in Table 1. Most of the cohort were female, with a median age of 56 years (30—83 years), and most of the primary tumors were lung and breast cancers, of which the top two were gene-driven non-small cell lung cancer (NSCLC) (44.7%) and Her-2 or BRCA-positive invasive ductal carcinoma of breast (9.7%). The median BM number was 2 (1–21) and the median BM volume was 8.4 cc (0.1–125.8 cc). Most of the patients had good KPS scores. The majority were untreated patients (68.3%). All patients received HFRT, 67 (54.5%) patients received concurrent or sequential chemotherapy, 56 (45.5%) patients received concurrent or sequential temozolomide (TMZ), 61 (49.6%) patients received concurrent or sequential targeted therapy, and 7 (5.7%) patients received sequential immunotherapy. All patients underwent MRI for simulation localization and contouring. Sixty-three patients underwent MRI during radiotherapy.

**Table 1:** Baseline clinical characteristics in the whole group of patients

Characteristic	N (%)
The median age, range (yrs)	56, 30–83
Gender	
Male	53 (43.1)
Female	70 (56.9)
Primary tumor	
NSCLC	73 (59.3)
SCLC	20 (16.3)
BC	22 (17.9)
Others	8 (6.5)
Gene mutation	
EGFR	47 (38.2)
ALK	8 (6.5)
Her-2	11 (8.9)
BRCA	1 (0.8)
None of above listed	56 (45.5)
BM location	
Cerebrum	93 (75.6)
Cerebellum	29 (23.6)
Brainstem	1 (0.8)
KPS	
≥ 80	94 (76.4)
< 80	29 (23.6)
BM volume	
≥ 6 cc	72 (58.5)
< 6 cc	51 (41.5)
RT regimens	
PCI or previous WBRT	33 (26.8)
HFRT alone	69 (56.1)
SRS concurrent with WBRT	21 (17.1)
Systemic treatments	
Chemotherapy	57 (46.3)
TMZ	56 (45.5)
Targeted therapy	61 (49.6)
Immunotherapy	7 (5.7)

*N* number, *BM* brain metastases, *EGFR* epidermal growth factor receptor, *ALK* anaplastic lymphoma kinase, *Her-2* Human epidermal growth factor receptor-2, *BRCA* Breast cancer susceptibility gene, *KPS* Karnofsky Performance Score, *RT* radiation therapy, *SRS* stereotactic radiosurgery, *TMZ* Temozolomide

### Radiation treatment

A total of 69 patients (56.1%) received HFRT alone, 21 patients (17.1%) received WBRT concurrent with region of interest (ROI) (SIB), 33 patients (26.8%) received WBRT followed by HFRT and 19 patients (15.4%) within 1 year (Additional file 2: Table S1). Of the 40 patients, 17 patients received WBRT with 2D, 5 patients with IMRT, 1 patient with VMAT and 18 patients with TOMO. The median prescribed dose of GTV was 52.5 Gy (20–94 Gy) and the median equivalent biological dose (BED) was 72.8 Gy (39–152 Gy) for the whole group of patients. The actual median Dmean was 57.4 Gy, Dmax was 61.9 Gy, the minimum dose (Dmin) was 46.3 Gy, and the V95 was 99.7%. Twenty-eight patients (22.8%) received boost area in ROI with median dose of 64.4 Gy. The Dmean of dose dropping zone was 53.9 Gy, V95 was 98.0%, 44 patients (35.8%) received field-shrinking during HFRT, and the median reduction rate of GTV was 31.2% (4.0–85.7%).

The average whole brain volume for the whole group was 1348.0 ± 147.2 cm^3^, and the average volume of normal brain tissue was 1329.5 ± 145.7 cm^3^. The dose parameters related to the target BM are shown in Table 2. The average hippocampus PRV was 17.1 ± 7.3 cm^3^, Dmax was 27.2 ± 22.1 Gy, and Dmean was 13.1 ± 14.7 Gy. The brainstem was contoured and limited in all patients, with the average brainstem volume of 26.4 ± 3.8 cm3, Dmax of 20.3 ± 20.2 Gy, and Dmean of 13.4 ± 15.1 Gy. The average volume of brainstem PRV was 42.7 ± 5.6 cm^3^, Dmax was 23.2 ± 22.1 Gy, and Dmean was 11.4 ± 15.2 Gy.

**Table 2:** Dosimetric parameters related to hyper-fractionated radiotherapy for the target lesion

Parameters	Median	Mean ± standard deviation	Minimum	Maximum
BED (Gy)	72.8	74.5 ± 15.5	39.0	152.0
TC (%)	95.7	93.9 ± 7.5	55.0	100.0
PITV	1.2	1.5 ± 1.0	0.7	10.2
CI	0.7	0.7 ± 0.2	0.1	1.0
HI	0.1	0.2 ± 0.1	0.0	0.6
GI	3.8	4.5 ± 1.0	0.7	10.2
Dose dropping zone Dmean (Gy)	53.9	51.1 ± 12.9	12.2	94.4
Dose dropping zone V95 (%)	98.0	89.5 ± 16.4	6.1	100.0
Normal brain tissue Dmean BED (Gy) in HFRT group	3.7	4.8 ± 3.9	0.2	17.4
Normal brain tissue Dmean BED (Gy) in WBRT + HFRT group	49.4	35.8 ± 21.8	2.5	59.8

*BED* Biological equivalent dose, *TC* Target coverage, *PITV* Prescription isodose target volume ratio, *CI* Conformity index, *HI* Homogeneity index, *GI* Gradient index, *Dmean* The mean dose, *V95* the volume that received 95% of the prescribed dose, *V30* the volume receiving 30 Gy irradiation

### Anti-angiogenic drug application

During the follow-up period, 63 patients (51.2%) were treated with the humanized-anti-VEGF monoclonal antibody bevacizumab (Avastin), including 23 patients (48.9%) in the TR group and 40 patients (52.6%) in the PP group. The median dose was 300 mg/m^2^/q21d *iv*, the median application cycles number was 2 (1–17), and the median interval between RT and drug administration was 13.7 months (- 0.7–91.6 months). The effectiveness rate was statistically significant different between the TR and PP groups (43.8% vs. 96.4%, *P* < 0.001), with 34 patients (54.0%) having a reduction in the size of lesions or edema area and obvious remission in neurological symptoms.

### Survival outcomes

The median follow-up time was 68.4 months (range, 8.9–146.2 months), and the median time from HFRT to the need for differential MRI evaluation was 14.0 months (IQR, 8.9–23.5 months). Long-term post-treatment follow-up brain MRI showed multiple dynamic changes in the treated lesions, including: shrinkage followed by enlargement (90 cases, 73.2%), enlargement followed by shrinkage (4 cases, 3.3%), delayed enlargement (12 cases, 9.8%), transient enlargement (9 cases, 7.3%), and constant enlargement (8 cases, 6.5%). Seventy-six patients (61.8%) were diagnosed with PP and 47 patients (38.2%) were diagnosed with TR (Additional file 1: Fig. S1), as showed by surgical pathology or diagnostic imaging consultation, which occurred in 18 patients (78.3%) and 5 patients (21.7%) in the validation cohort. The median time from RT to PP was 18.4 months (IQR, 9.4–28.5 months), and the median time from RT to TR was 12.5 months (IQR, 8.3–19.3 months). In this enrolled patient population, the 1-year local tumor control rate was 84.4%.

After the fusion of the review MRI images with the radiotherapy planned images, defining the tumor margin as an area of 2 mm 3D-outgrowth of GTV, the results revealed that in the TR group, recurrence sites included: infield (n = 15, 31.9%), margin (n = 23, 48.9%) and outfield (n = 9, 19.1%), with a median previously irradiated dose of 45 Gy in the recurrence area (10–60 Gy).

### Salvage Treatment

**Fig. 2:**
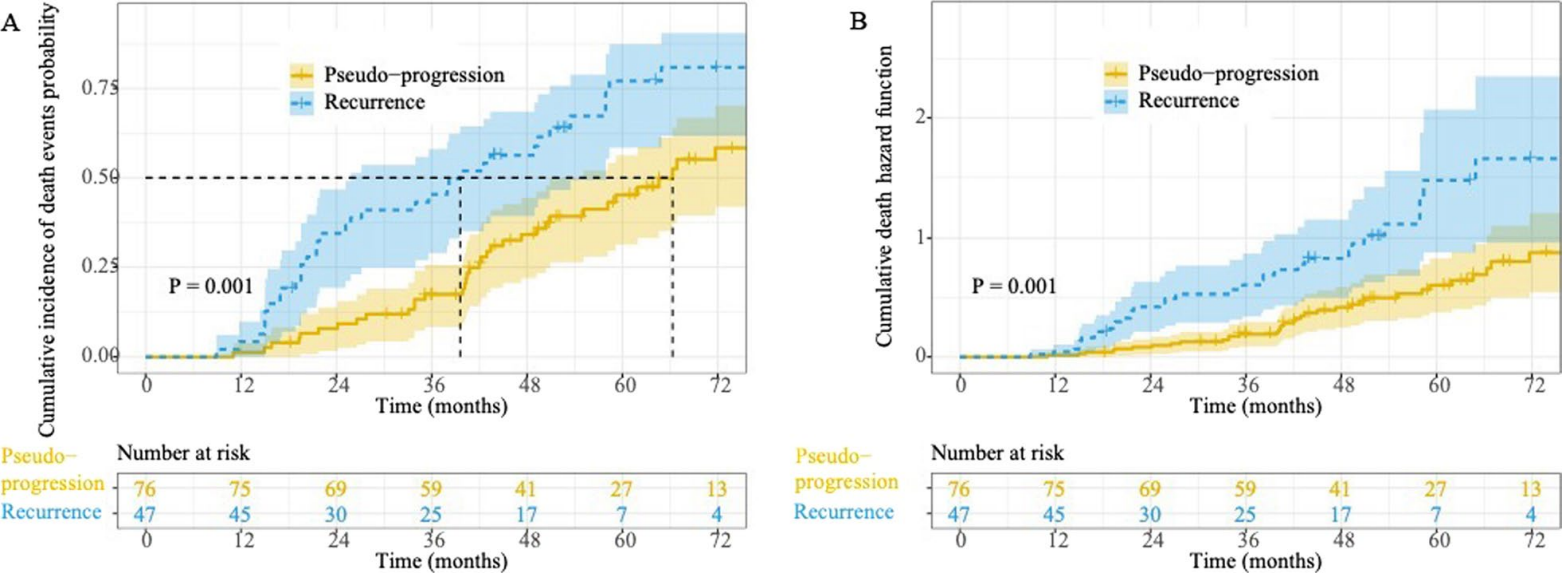
Kaplan–Meier method to calculate the cumulative incidence of death (**A**) and risk function (**B**) for different groups of patients

A total of 57 patients (46.3%) in the whole group and 31 patients (66.0%) in the TR group received salvage treatment, including salvage RT (n = 34, 27.6%), surgical resection (n = 21, 17.1%), or combined systemic chemotherapy (n = 12, 9.8%). Ten patients (17.5%) had more than one salvage treatment. The 1-, 2-, and 5-year survival rates for the entire group were 97.6%, 82.0%, and 42.7%, respectively. The median survival times were 39.6 months (95% CI 25.4–53.8 months) and 66.3 months (95% CI 58–75.9 months) for the TR and PP groups, respectively, with a statistical difference in the cumulative risk of death (*P* = 0.001; Fig. 2). A total of 73 patients died during follow-up, with the main causes of death including BM (n = 37, 50.7%), systemic failure (n = 18, 24.7%), extracranial tumor (n = 13, 17.8%), and medical comorbidities (n = 5, 6.8%). Specific causes of death for BM included uncontrolled target lesions (n = 11, 29.7%), uncontrolled cerebral edema (n = 4, 10.8%), newly diagnostic BM (n = 5, 13.5%), leptomeningeal metastases (n = 14, 37.8%), ruptured bleeding from BM (n = 2, 5.4%), and progressive dementia (n = 1, 2.7%).

### Nomogram prediction model and validation

**Fig. 3:**
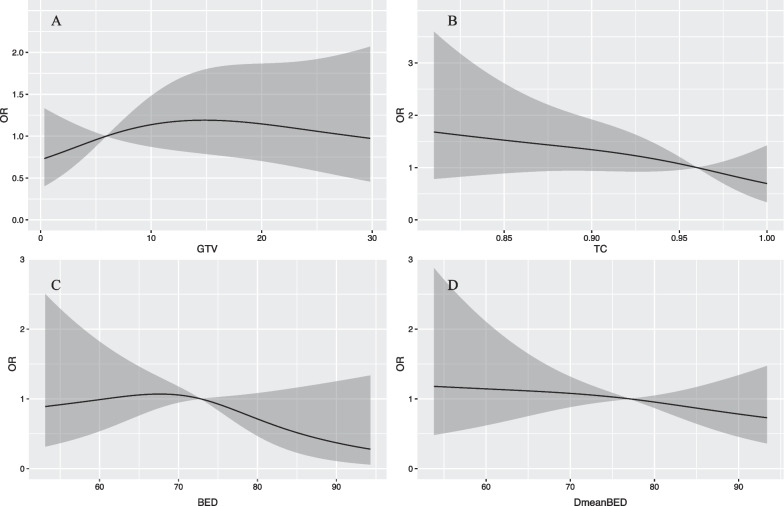
Restricted cubic spline curves of recurrence hazard ratio for target lesion volume (**A**), target area coverage (**B**), equivalent biological dose (**C**) and average dose in the dose dropping zone (**D**)

Logistic regression models (backward approach) were used to screen for factors that might be associated with local tumor recurrence, including: GTV volume, treatment modality, whether to receive field-revision during treatment, prescription BED, dose dropping zone Dmean BED, TC, whether to receive targeted therapy, and whether to receive chemotherapy. The restricted cubic spline function was used to find the optimal cut-off values for the continuous variables: 6 cc and 28 cc for GTV volume, 60 Gy and 73 Gy for BED, 0.96 for TC, and 77 Gy for the dose dropping zone Dmean BED (Fig. 3).

**Fig. 4:**
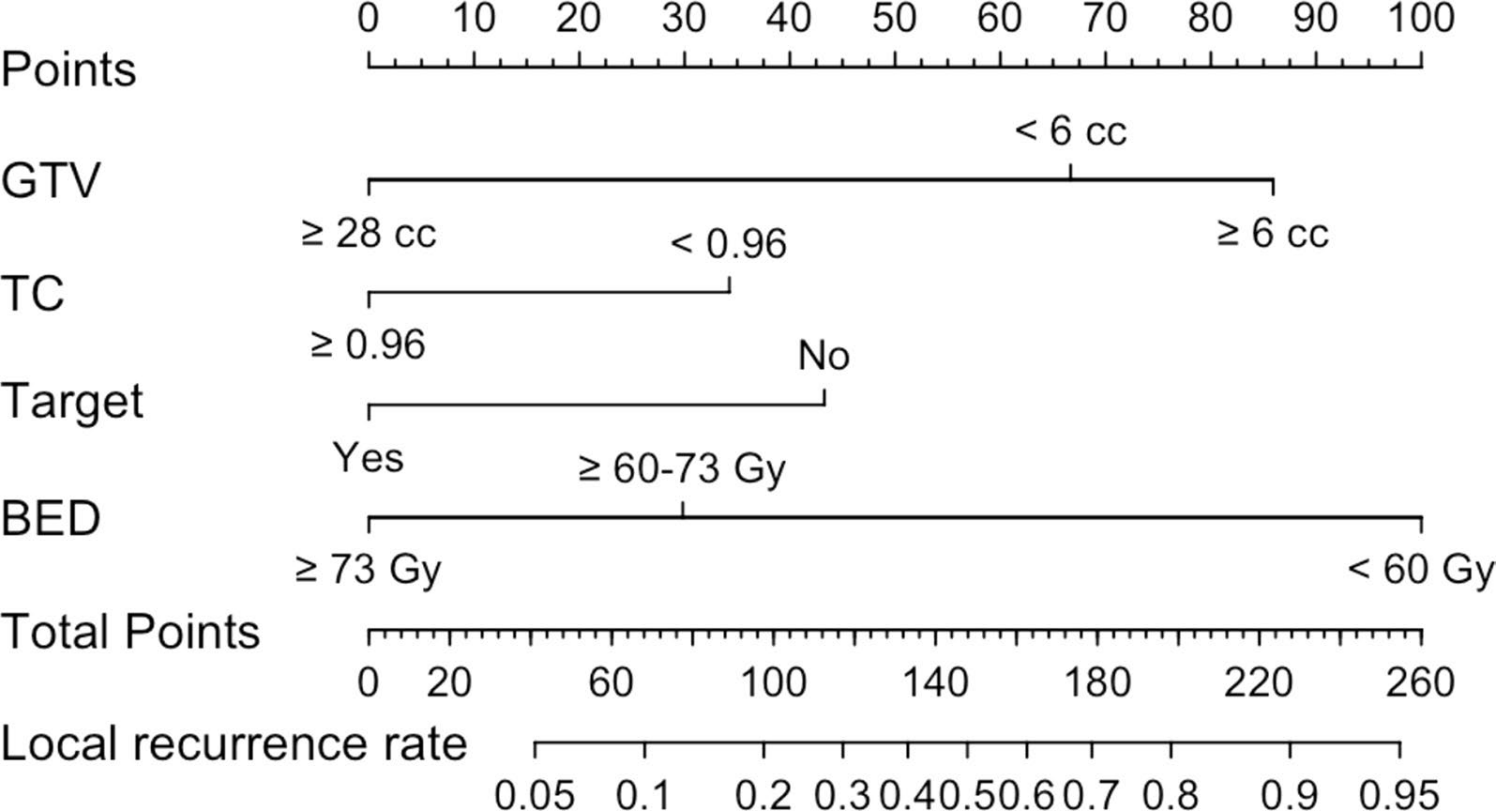
Nomogram prediction model for tumor recurrence in brain metastases

**Fig. 5:**
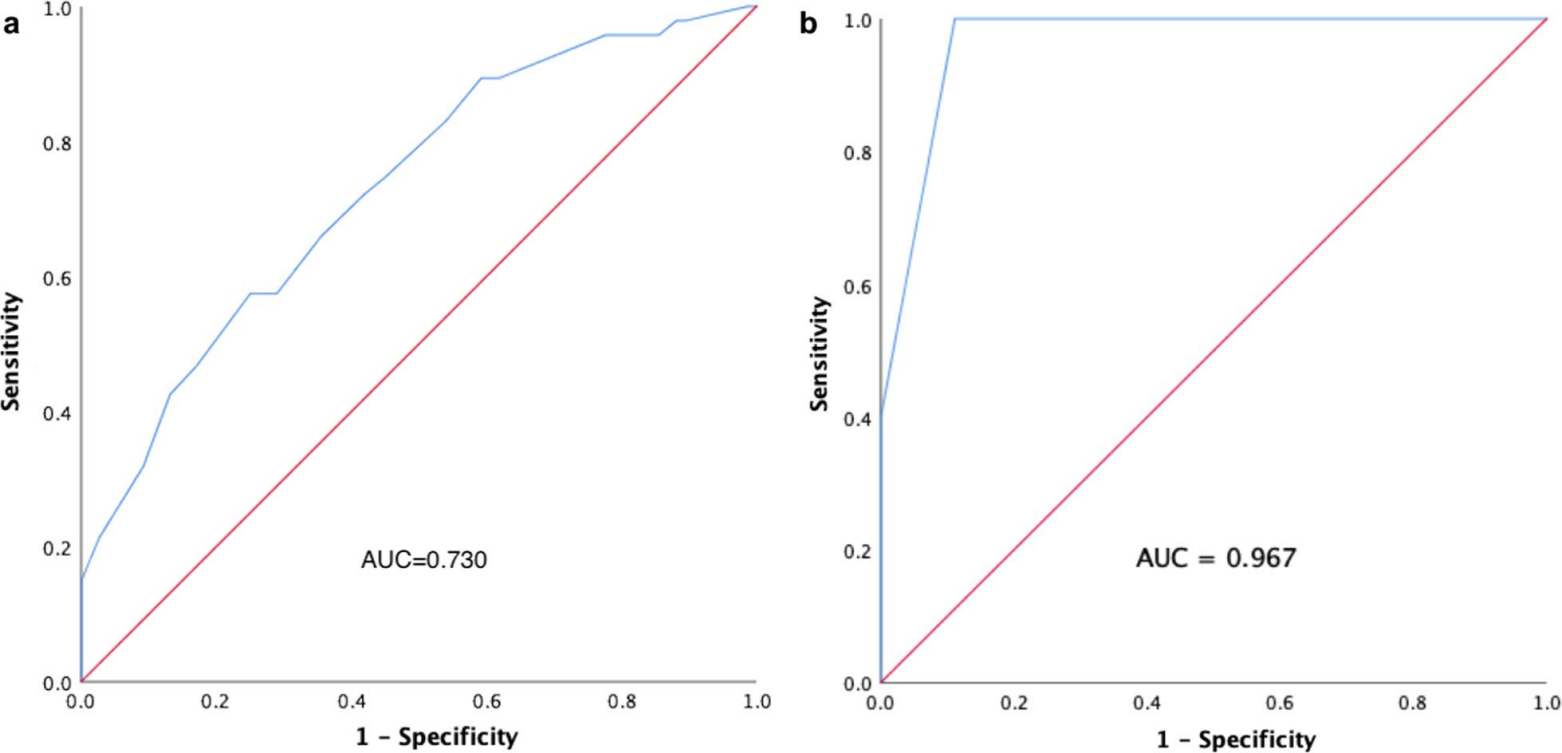
ROC curve and area under the curve (AUC) of the prediction model in the training cohort (**a**) and in the validation cohort (**b**)

**Fig. 6:**
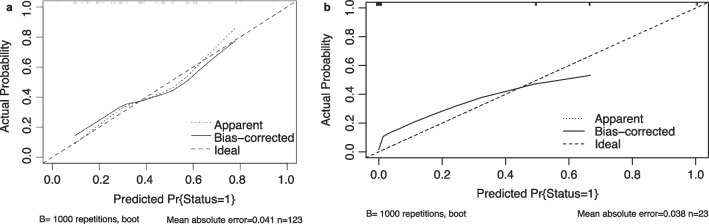
Calibration-correction curve of the prediction model in the training cohort (**a**) and in the validation cohort (**b**)

**Fig. 7:**
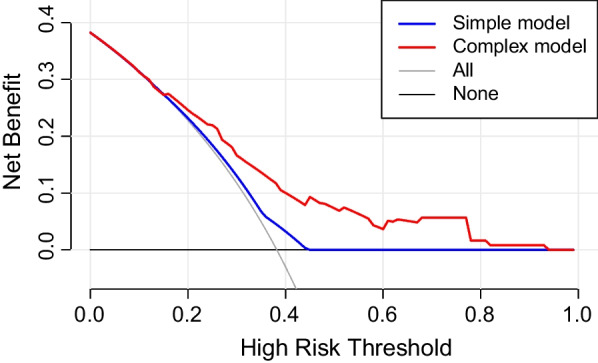
Decision curve analysis evaluating the prediction model

The multiple logistic regression analysis showed that GTV volume < 6 cc (vs. ≥ 6 cc: OR 1.65, 95% CI 0.70–3.87, *P* = 0.251; vs. ≥ 28 cc: OR 0.12, 95% CI 0.01–1.26, *P* = 0.077), BED < 60 Gy (vs. 60–73 Gy: OR 0.13, 95% CI 0.01–1.72, *P* = 0.120; vs. ≥ 73 Gy: OR 0.04, 95% CI 0.00–0.59, *P* = 0.019), TC ≥ 96% (vs. < 96%: OR 0.32, 95% CI 0.13–0.77, *P* = 0.011), prior targeted therapy (vs. no targeted therapy: OR 0.30, 95% CI 0.12–0.72, *P* = 0.007) were factors associated with TR. Bootstrap repeated sampling was performed to validate the above results (Table 3), and those with *P* < 0.050 were included in the Nomogram prediction model. The risk scores were calculated separately for each independent risk factor, and the probability of local tumor recurrence in BM was predicted based on the final score at the bottom (Fig. 4). The C-index according to this model, also known as the area under the ROC curves (AUC), were 0.730 in the training cohort and 0.967 in the validation cohort, respectively (Fig. 5a, b). A total of 1000 patients underwent field-revision during treatment, and the calibration-corrected curve prediction overlapped well with the actual probability, with a mean absolute error of 0.041 in the training cohort and 0.038 in the validation cohort (Fig. 6a, b). The decision curve analysis (Fig. 7) showed that in the approximate range of 0.1 to 0.8, the net benefit rate of the complex model (the prediction model) was higher than that of the simple model (only GTV included as the predictor). And the clinical impact curve also presented the difference between patients who were classified as high risk of TR by the simple and complex model under each threshold probability (Additional file 1: Fig. S2).

**Table 3:** Logistic regression screening for variables associated with the occurrence of tumor recurrence after hyper-fractionated radiotherapy in patients with brain metastases

Variables	Original database			Bootstrap repeated sampling (n = 1000)		
	OR	95% CI	*P*	Standard error	95% CI	*P* (bilateral)
GTV (cc)						
< 6	1			1		
6–28	1.65	0.70–3.87	0.251	0.62	− 0.94–1.46	0.694
≥ 28	0.12	0.11–1.26	0.077	9.72	− 23.13–0.26	0.041
BED (Gy)						
< 60	1			1		
60–73	0.13	0.01–1.72	0.120	11.30	− 24.13 –18.61	0.027
≥ 73	0.39	0.00–0.59	0.019	11.26	− 25.93–16.95	0.003
TC						
≥ 0.96 vs. < 0.96	0.32	0.13–0.77	0.011	0.63	− 2.78 to − 0.23	0.013
Dose dropping zone Dmean (Gy)						
≥ 77 vs. < 77	2.34	0.92–5.99	0.075	0.66	− 0.15–2.44	0.090
Target therapy						
Yes vs. No	0.30	0.12–0.72	0.007	0.62	− 2.61 to − 0.12	0.028

*GTV* gross tumor volume, *BED* biological equivalent dose, *TC* target coverage, *Dmean* the mean dose, *OR* odds ratio, *CI* confidence interval

## Discussion

The results of this study show that clinical and dosimetric features analysis of RT plan based on contrasted brain MRI can help identify clinical outcomes after HFRT for patients with BM. Target area volume, BED, target area coverage and the presence of previous targeted therapy were associated with the occurrence of post-treatment TR and PP. The nomogram showed good prognostic efficacy and high generalizability when validated in an internal cohort.

For patients treated with SRS, the incidence of RN ranges from 5 to 25% [12]. Several previous studies have explored factors associated with the occurrence of PP after SRS for BM, broadly including clinical characteristics and RT dose parameters. The results of most of the previous studies suggest that lesion maximum diameter and target area volume are important risk factors for RN. Sneed et al. showed that in patients with BM without previous SRS, patients with lesion maximum diameter > 2.1 cm, target area volume > 1.2 cm^3^, prescription isodose line volume > 1.8 cm3,V12 Gy > 3.3 cm^3^, V10 Gy > 4.3 cm^3^, the incidence of symptomatic RN at 1 year after treatment was 13–14% [13]. Another meta-analysis showed that if BM received V20 Gy (3 f) and V24 Gy (5 f) < 20 cm^3^, the incidence of brain necrosis was < 10% and less than 4% of patients required surgical resection [14]. The clinical outcomes of the lesion also correlated with the dose and segmentation pattern of RT. It has been demonstrated that HFSRT is overall less likely to result in PP and grade IV RN requiring surgical resection than single SRS and does not increase the risk of intracranial progression [14, 15]. Patients would have a higher risk of RN in the 27 Gy/3 f group (HR 3.07, 95% CI 1.13–8.36, *P* = 0.030) and 35 Gy/5 f group (HR 4.22, 95% CI 1.46–12.21, *P* < 0.010) than in the 30 Gy/5 f group [16]. In another recent dosimetric study, the normal brain tissue volumes receiving 25 Gy and 30 Gy (BrainV25, BrainV30) were the most significant influences associated with symptomatic RN, patients with BrainV25 > 16 cm^3^ (HR 11.7[1.47–93.3]) had a 2-year incidence of symptomatic radionecrosis of 21% versus 2%, compared with BrainV30 > 10 cm^3^ (HR 7.08 [1.52–33.0]; *P* = 0.007) [17]. A meta-analysis showed that single SRS of BM receiving D12 Gy > 5 cm^3^, 10 cm^3^ and > 15 cm^3^ were associated with 10%, 15% and 20% of symptomatic RN [14].

In addition, some studies have shown that tumor maximum diameter, previous WBRT, prescribed dose, and primary tumor type were associated with PP in univariate analysis, but only tumor maximum diameter (HR 3.10, *P* < 0.001) remained statistically significant in multivariate analysis [18]. The results of the Norwegian study showed that previous WBRT or SRS, TC ≥ 98%, BM volume ≤ 2 cm3, and primary lung cancer were influential factors associated with PP, and a prediction model was established with 100% PP when < 2 points, 57% PP when 2 points, 43% PP when 3 points, and 16% PP when > 3 points [19]. Therefore, the prognostic regression of patients can now be initially judged according to the baseline characteristics and parameters, such as RT dose parameters.

The results of this study suggest that clinical outcomes are related to the BED of RT prescription and TC received by the lesion. Patients with higher BED and TC are more likely to experience PP, and patients with GTV volume ≥ 6 cc were more likely to experience TR than smaller lesions. Those with target area volume ≥ 28 cc may have chosen a more intense treatment regimen due to heavier intracranial tumor burden (received BED < 60 Gy: 6.5% vs. 0.0%, *P* = 0.049), so it was instead positively associated with the occurrence of PP compared to patients with small lesions. In addition, considering that patients in this study received individualized segmentation patterns, there was no direct correlation between clinical outcomes and the specific dose patients received. The primary tumor type and radiotherapy modality were also not associated with outcomes, probably because the prescribed dose for BM was not set according to the primary tumor type. Moreover, more than half of the patients were gene-driven positive NSCLC (54/73, 74.0%) and Her-2 or BRCA-positive breast cancer (13/22, 59.1%) who could receive targeted therapy, which could have influenced clinical outcomes especially on intracranial and extracranial tumor control.

In addition to local RT, systemic therapy agents received by patients may also cross the blood–brain barrier and have an effect on BM. Multivariable analysis of a previous study showed that oral capecitabine was associated with the occurrence of PP, in addition to volume > 1.0 cm^3^, previous SRS, primary renal tumor and connective tissue disease [11]. The results of this study showed that having received targeted therapy before the identification of MRI was a protective factor for TR, while the effect of chemotherapy and immunotherapy did not reach a statistical difference. First, about 20% (23/123) of patients received concurrent or sequential targeted therapy with chemotherapy, which might indicate that there was an interaction between the effects of chemotherapy and targeted therapy, and the effect of chemotherapy on the outcome was underestimated; Second, less than half of the cases in this study received systemic chemotherapy and immunotherapy, while many patients had intracranial controlled but primary and extracranial metastases progressed and received salvage treatment. However, in the PP group, chemotherapy was often administered after primary tumor or extracranial metastases progressed, and 24% (18/76) of patients had received chemotherapy within 6 months before the incidence of PP.

In addition, several studies aimed to distinguish PP between TR after SRS by functional MRI. The analysis of magnetic resonance perfusion imaging revealed reduced relative cerebral blood volume (rCBV) values in non-progressive, PP and indeterminate lesions, while progressive lesions showed increased rCBV values [20]. Similarly, TR was shown to be elevated by relative standardized uptake value (rSUV) in F-DOPA PET (accuracy = 94.1% for an optimal threshold of 1.92), but tumor size did not differ between progressive and necrotic lesions [21]. In recent years, methionine-labeled (11C-methionine PET, MET-PET) has also been reported to identify TR versus PP in patients with BM [22].

However, considering the financial conditions of patients and the limited medical resources, the most widely used method for post-treatment evaluation of BM is still normal brain MRI. Imaging studies of PP have focused on extracting numerous features from the texture, shape and heterogeneity of brain MRI to differentiate from TR. The results showed that the features extracted from FLAIR sequences were the most discriminatory, with AUC values of 0.79 ± 0.09 (95% CI 0.75–0.83); and accuracy of 0.75 ± 0.06 (95% CI 0.72–0.78) [23]. In another study, 282 radiomic features were extracted from two different scan time points that could distinguish between true and pseudo-progression, and the model was narrowed down to 11 features by cross-validation, with an AUC value of 0.79 and discriminatory accuracy of 83% [24]. In this study, we identified TR and PP based on enhanced brain MRI, and the results showed that most of the BM recurrence was at marginal area of the original treated lesions with a median dose of 45 Gy, which suggests that oncologists should pay attention to the dose distribution of the dose dropping area. In addition, BM volume after HFRT showed various types of dynamic changes, suggesting that we should not evaluate the post-treatment efficacy only on the increase of the treated lesions, but also focus on the change pattern of lesions over time and the evaluation of their response to dehydrating agents and anti-vascular survival drugs during follow-up. However, this study only summarized the type of dynamic changes of imaging types, BM and peritumoral edema volume, as well as the specific recurrence patterns and median dose received. We will further analyze the imaging data, especially by combining radiomics and deep learning methods, to explore the brain MRI features that distinguish TR from PP to improve the accuracy and practical application value of this model.

According to the National Cancer Institute Common Terminology Criteria for Adverse Effects (NCI-CTCAE) for RN, patients with grade III edema, necrosis, and cystic lesions are currently treated with dehydrating agents, corticosteroids, and anti-vascular drugs to control neurological signs and symptoms, but hormonal therapy is not indicated for patients with residual or recurrent tumors and patients with high risk of hormonal side effects. Once a grade IV diagnosis is diagnosed, surgical excision of the lesion and surrounding edematous areas should be considered to immediately relieve cranial hypertension. Persistent diffusion restriction may occur in patients with PP and RN, and the vascular endothelial growth factor inhibitor bevacizumab is effective in controlling the edema and mass effect [25–27]. The results of a meta-analysis showed that when bevacizumab was administered, imaging shrinkage was observed in 93% of patients, with stabilization in 10%, improvement in 48%, and complete remission in 40% of symptomatic patients [8]. There was no significant difference between the regimens such as 5 vs. 10 mg/kg, 2 vs. > 2 weeks in terms of cerebral edema relieving effect. Therefore, the current principles for the administration of anti-vascular drugs mainly recommend small doses of periodic intravenous injections [28]. In this study, the anti-vascular endothelial growth factor (VEGF) monoclonal antibody bevacizumab was administered to more than half of the patients, mostly for the treatment of intractable cerebral edema resistant to dehydration agents and a minority for sequential chemotherapy. There was a statistically significant difference in efficacy between the two groups, with a higher rate of neurological symptom remission in the PP group and a significantly shorter duration of remission in the case of TR, which might help to identify clinical outcomes.

## Conclusion

GTV, BED, TC and whether the patient previously received targeted therapy are the relevant factors affecting the outcomes. The established nomogram prediction model may help to distinguish PP from TR after HFRT for BM.

## Supplementary Information

**Additional file 1. Fig. S1–2.** Examples of RT planning and clinical impact curve.

**Additional file 2. Table S1.** The region of interest of target area.

## Data Availability

The datasets analyzed during the current study are available from the corresponding author on reasonable request.
