# Supplementary material for: Dosimetric and clinical analysis of pseudo-progression versus recurrence after hypo-fractionated radiotherapy for brain metastases
Source: Radiat Oncol. 2023 Feb 14;18:30. doi: 10.1186/s13014-023-02214-7 (PMC9930329; doi:10.1186/s13014-023-02214-7)
Supplement: Supplementary file 1 — Additional file 1. Fig. S1–2. Examples of RT planning and clinical impact curve. [file 13014_2023_2214_MOESM1_ESM.docx]

Dosimetric and clinical analysis of pseudo-progression vs. recurrence after hypo-fractionated radiotherapy for brain metastases

Siran Yang^1,3,a^, Yuchao Ma^1,a^, Yingjie Xu^1^, Qingfeng Liu^1^, Ye Zhang^1^, Xiaodong Huang^1^, Xuesong Chen^1^, Kai Wang^1^, Yuchao Ma^1^, Ruizhi Zhao^1^, Jianping Xiao^1*^, Hongmei Zhang^2*^

^1^Departments of Radiation Oncology, National Cancer Center/National Clinical Research Center for Cancer/Cancer Hospital, Chinese Academy of Medical Sciences and Peking Union Medical College, Beijing, People’s Republic of China

^2^Departments of Diagnostic Radiology, National Cancer Center/National Clinical Research Center for Cancer/Cancer Hospital, Chinese Academy of Medical Sciences and Peking Union Medical College, Beijing, People’s Republic of China

^3^Department of Radiation Oncology, Peking University Shenzhen Hospital, Shenzhen, People’s Republic of China

*** Correspondence:**

Jianping Xiao

[jpxiao8@163.com](mailto:jpxiao8@163.com)

Hongmei Zhang

[13581968865@163.com](mailto:13581968865@163.com)

*^a^ Siran Yang and Yuchao Ma contributed equally to this work.*

Fig. S1 Examples of brain metastases at planning system before treatment and pseudo-progression (A) and tumor recurrence (B) on magnetic resonance imaging after hyper-fractionated radiotherapy

Fig. S2 Clinical impact curve^*^ that evaluating the difference between the simple model (A) and the complex model (B) for appropriate use

^*^The DCA curve uses simple and complex models to predict risk stratification for 1000 people, respectively, and the abscissa of the graph is Threshold Probability. When various evaluation methods reach a certain value, the probability of death risk of patient i is recorded as Pi; When pi reaches a certain threshold (denoted as Pt), it is defined as positive and some kind of intervention is taken. The ordinate is the net rate of benefit after subtracting the disadvantage. The y-axis is the calculated payoff, and the x-axis is the value of the different probabilities Pt.
