# Supplementary material for: Dosimetric and clinical analysis of pseudo-progression versus recurrence after hypo-fractionated radiotherapy for brain metastases
Source: Radiat Oncol. 2023 Feb 14;18:30. doi: 10.1186/s13014-023-02214-7 (PMC9930329; doi:10.1186/s13014-023-02214-7)
Supplement: Supplementary file 2 — Additional file 2. Table S1. The region of interest of target area. [file 13014_2023_2214_MOESM2_ESM.docx]

Dosimetric and clinical analysis of pseudo-progression vs. recurrence after hypo-fractionated radiotherapy for brain metastases

Siran Yang^1,3,a^, Yuchao Ma^1,a^, Yingjie Xu^1^, Qingfeng Liu^1^, Ye Zhang^1^, Xiaodong Huang^1^, Xuesong Chen^1^, Kai Wang^1^, Yuchao Ma^1^, Ruizhi Zhao^1^, Jianping Xiao^1*^, Hongmei Zhang^2*^

^1^Departments of Radiation Oncology, National Cancer Center/National Clinical Research Center for Cancer/Cancer Hospital, Chinese Academy of Medical Sciences and Peking Union Medical College, Beijing, People’s Republic of China

^2^Departments of Diagnostic Radiology, National Cancer Center/National Clinical Research Center for Cancer/Cancer Hospital, Chinese Academy of Medical Sciences and Peking Union Medical College, Beijing, People’s Republic of China

^3^Department of Radiation Oncology, Peking University Shenzhen Hospital, Shenzhen, People’s Republic of China

*** Correspondence:**

Jianping Xiao

[jpxiao8@163.com](mailto:jpxiao8@163.com)

Hongmei Zhang

[13581968865@163.com](mailto:13581968865@163.com)

*^a^ Siran Yang and Yuchao Ma contributed equally to this work.*

Table S1 The region of interest of target area for patients received hypo-fractionated radiotherapy in the study

| ROI | Numbers |
| --- | --- |
| WBRT concurrent with PTV | 3 |
| WBRT concurrent with GTV | 11 |
| WBRT concurrent with GTV+Boost | 7 |
| WBRT followed by PTV | 10 |
| WBRT followed by GTV | 5 |
| WBRT followed by GTV+Boost | 4 |
| PTV | 60 |
| GTV | 6 |
| PTV+GTV | 1 |
| PTV+Boost | 7 |
| GTV+Boost | 6 |
| PTV+GTV+Boost | 3 |
| Total | 123 |

Abbreviation: ROI, region of interest; PTV, planned target volume; GTV, gross target volume.
